# The role of early attachment experiences in modulating defensive peripersonal space

**DOI:** 10.1038/s41598-023-30985-2

**Published:** 2023-03-07

**Authors:** Carlotta Fossataro, Mauro Adenzato, Margherita Bruno, Elena Fontana, Francesca Garbarini, Rita B. Ardito

**Affiliations:** 1 MANIBUS Lab, Department of Psychology, University of Turin, Via Verdi 10, 10124 Turin, Italy; 2 Department of Psychology, University of Turin, Turin, Italy; 3 Neuroscience Institute of Turin (NIT), Turin, Italy

**Keywords:** Psychology, Human behaviour

## Abstract

Selecting appropriate defensive behaviours for threats approaching the space surrounding the body (peripersonal space, PPS) is crucial for survival. The extent of defensive PPS is measured by recording the hand-blink reflex (HBR), a subcortical defensive response. Higher-order cortical areas involved in PPS representation exert top-down modulation on brainstem circuits subserving HBR. However, it is not yet known whether pre-existing models of social relationships (internal working models, IWM) originating from early attachment experiences influence defensive responses. We hypothesized that organized IWM ensure adequate top-down regulation of brainstem activity mediating HBR, whereas disorganized IWM are associated with altered response patterns. To investigate attachment-dependent modulation on defensive responses, we used the Adult Attachment Interview to determine IWM and recorded HBR in two sessions (with or without the neurobehavioral attachment system activated). As expected, the HBR magnitude in individuals with organized IWM was modulated by the threat proximity to the face, regardless of the session. In contrast, for individuals with disorganized IWM, attachment system activation enhances HBR regardless of the threat position, suggesting that triggering emotional attachment experiences magnifies the threatening valence of external stimuli. Our results indicate that the attachment system exerts a strong modulation on defensive responses and the magnitude of PPS.

## Introduction

In the course of our life, we develop a neural system that is specifically responsible for representing the space immediately close to the body: the peripersonal space (PPS)^1,2^. The PPS is a multisensory representation of the space surrounding the body that uses the body itself as a reference to encode both nearby objects and approaching threats. In a threatening context, the PPS is used to protect the body. The closer to the body a threat occurs, the more likely it is to cause harm, thus reinforcing our defensive and avoidance responses^3–5^. Therefore, an accurate representation of this part of the space, namely the defensive PPS, and continuous monitoring of environmental threats approaching the body is essential for survival.

To measure the extent of people’s defensive PPS and the magnitude of their defensive responses, a widely used parameter is the hand blink reflex (HBR), a subcortical defensive response known to be modulated by the position of the threat in relation to the PPS of the face^6^. HBR is elicited by electrical stimulation of the median nerve at the wrist and recorded by electromyography (EMG) at the orbicularis oculi. The magnitude of HBR is significantly increased when the threatened hand, receiving the electrical stimulation, is close to the participants’ face as compared to when the hand is stimulated far from the face^6–14^. The position at which the first HBR enhancement can be observed in comparison to the far position delimits the imaginary boundary of the PPS of the face within which approaching threats are perceived as more threatening the closer they are. Specifically, if we conceive the PPS as a defensive cloud surrounding the face, the modulation of HBR magnitude as a function of hand position in space (i.e., far, near, ultra near) can be used to map the extent of the PPS boundaries. Indeed, geometric modelling of HBR enhancement led to define the defensive PPS anchored to the face as a bubble elongated along the vertical axis^15^. The boundary of PPS (which, as noted above, is conceptualized as the position of the first robust HBR magnitude increase at group level) is located between 20 and 40 cm from the face, and within this space HBR responses are further enhanced with proximity of the threat to the face, defining a high-risk area^15,16^. Therefore, participants who show an abrupt enhancement of HBR magnitude at the nearest distance to the face (i.e., ultra near) have a smaller PPS extent than participants who already show a clear enhancement at a greater distance (i.e., near position).

It is known that neural circuits at the level of the brainstem mediate HBR and that these circuits are interconnected with higher-order cortical areas involved in the representation of PPS^6^. Interestingly, personality factors such as empathic^7^ and anxious^16^ traits contribute to individual variability in both the extent of defensive PPS and the magnitude of defensive responses. This is consistent with the observation that subcortical neural circuits in the brainstem can undergo significant top-down modulation by the neocortex^8^.

Given these premises, in the present work we hypothesize that another dimension that could modulate the extent of defensive PPS and the magnitude of defensive responses is attachment. According to the attachment theory^17–19^, humans have an innate biological predisposition to seek the proximity of a caregiver who can provide protection and care when the need for it is aroused, for example, by fear or pain^20–23^. Bowlby attributes a protective function to this neurobehavioral system. According to this author, the need for care triggered by activation of the attachment system tends to elicit complementary caring behavior on the part of the caregiver. As a result of ongoing interactions with caregivers, each child structures interpersonal cognitive schemas or internal working models (IWM). IWM are mental representations of an individual’s attachment experiences in childhood that are formed from repeated interactions between the child and the caregiver (often the mother in the early years) and that usually remain stable into adulthood^24–29^. The main function of IWM is to predict what can be expected from the world and from interaction with it.

Depending on the nature of parental care, the structure of mental representations of attachment experiences in childhood (i.e., the IWM) may be organized or disorganized. When the structure of the IWM is organized, people develop coherent strategies when asking for care^26,30,31^ and have coherent mental states when asked to recall attachment in adulthood^32^. In contrast, when the structure of the IWM is disorganized, people have disoriented and/or disorganized memories of trauma or loss, incoherent expectations of attachment figures, and inconsistent behavioral strategies when asking for care^32^. Disorganized IWM are associated with frightening and threatening parenting styles^33^.

Interestingly, the neurobehavioral system of attachment has been described as part of a hierarchically organized control system in which the orbitofrontal cortex, anterior cingulate, and amygdala play the role of interconnected hubs that can regulate the activity of brainstem and midbrain nuclei^34,35^. Converging evidence^36–38^ suggests that in people with organized IWM these hubs exert control in regulating brainstem response to environmental stimuli and promote the generation of adaptive responses. On the contrary, in people with disorganized IWM, this top-down control is much less efficient and brainstem responses to environmental stimuli are under-modulated or dysregulated.

To our knowledge, the role of mental representations related to childhood attachment experiences (i.e., IWM) in modulating PPS extent and defensive responses has never been investigated, and we aim to systematically examine this here. To this end, we used the Adult Attachment Interview (AAI)^39^. The AAI is based on Bowlby’s attachment theory^17–19^ and is an interview that focuses on early attachment experiences and their effects and can be used to determine whether a person has organized or disorganized IWM. Previous findings^40,41^ show that in individuals classified as having organized IWM a level of organized strategies and integrative processes is maintained during activation of attachment-related memories, whereas in individuals classified as having disorganized IWM, activation of attachment-related memories could lead to a temporary failure of brain connectivity and a consequent disruption of high integrative mental functions. Furthermore, since the neurobiological attachment system exerts its own regulatory effects on brainstem activity mainly when activated, in the present study we also used an instrument known to act as a trigger of the attachment system, namely the Adult Attachment Projective (AAP). The AAP^42–45^ is a semi-structural interview that combines projective and interview techniques. The ability of the AAP to trigger attachment system has been confirmed in several research studies^46–50^. Therefore, we used the AAI to classify participants’ IWM and the AAP to experimentally trigger the neurobiological attachment system.

For all the above reasons, we hypothesize here that individuals with organized IWM have adequate top-down regulation of the activity of the neural circuits at the level of the brainstem that mediate HBR, and therefore will show a linear trend of HBR enhancement as the distance between the hand and the face decreases in the experimental setting. In contrast, we hypothesize that individuals with disorganized IWM will show an altered pattern of HBR responses, indicating dysregulation of control exerted on brainstem activity by higher-order structures associated with the attachment system.

## Materials and methods

### Participants

Thirty-two healthy participants were recruited for the study, and 26 of them (18 self-identifying females and 8 self-identifying males, mean age ± SD = 21.11 ± 1.21) were HBR responders and thus engaged for the study. Exclusion criteria were: (1) left-handedness, (2) history of neurological and/or psychiatric disorders, (3) head trauma, (4) use of medications affecting the central nervous system in the 2 weeks prior to participation in the experiment.

The percentage of HBR responders (81%) was consistent with previous studies^6,8,14^. The sample size was chosen based on previous HBR studies with healthy participants^7,51^. In these studies, the sample size ranged from a minimum of 13 to a maximum of 20 participants. In the current study, because we needed to divide the sample into two groups based on the AAI classification system (i.e., Organized and Disorganized), we included a sample of 26 participants. All participants were naïve about the experimental procedure and the purpose of the study, and gave written informed consent to participate in the study. In accordance with the Declaration of Helsinki (BMJ 1991; 302: 1194), all experimental procedures were approved by the Ethics Committee of the University of Turin (Prot. n. 117174).

### Experimental procedure

#### Adult attachment interview

To divide the sample into individuals with organized IWM and individuals with disorganized IWM, we used the AAI of George, Kaplan, and Main^39^. As many studies have shown (e.g.,^52–55^), the AAI is the instrument with greater reliability and validity for examining adult attachment and corresponding IWM. The interview classifies a person’s “state of mind” in relation to his or her attachment experiences in childhood and allows one to understand the processes that characterize the development of attachment experiences from the first months of life into adulthood.

The AAI is conducted by a trained clinical psychologist in a quiet and comfortable room. The interview lasts an average of one hour and 30 min and consists of a sequence of questions asked in a fixed order aimed at exploring the degree of integration between episodic and semantic memory while the person retrieve childhood (0–12 years old) emotional and relational memories concerning his or her parents, moments of vulnerability (separation, rejection), and, if present, experiences of abuse and loss. In addition, the person is asked to evaluate early attachment experiences and current attachment relationships, and to reflect on present or future dimensions of parenting. The entire interview is recorded, then transcribed, and finally coded using a scoring and classification system^56^. This system is based on the person’s ability to produce a coherent narrative, actively collaborate with the interviewer, and engage in “metacognitive monitoring” while recalling and describing his or her childhood memories. The result is the identification of three main categories of states of mind related to attachment: Free, Dismissing and Entangled. *Free* interviewees engage in coherent and collaborative discourse while describing and evaluating their attachment-related experiences, whether described as positive or negative. *Dismissing* interviewees do not support or contradict a normalizing and positive description of their parents with memories of specific events. All effects of negative experiences are denied. *Entangled* interviewees seem preoccupied, but may also be angry, confused or passive about the experiences they are reporting. In these three categories, although in very different ways, individuals demonstrate the presence of a strategy such that their IWM are considered as organized.

When a traumatic event (loss, physical or sexual abuse) has occurred and the narrative shows a lack of cognitive monitoring and elements of incoherence, the Unresolved category is added. If there is no strategy in the narrative and the state of mind fluctuates between Entangled and Dismissing, the category of Cannot Classify is applied. Individuals recognized as Unresolved and/or Cannot Classify are considered to have disorganized IWM.

#### Adult attachment projective

The AAP^42–45^ combines projective and interview techniques. Stability, predictive validity, and time efficiency are characteristics that make AAP one of the widely used instruments in both clinical and research settings^54^.

The interview takes place in a quiet room and lasts an average of 30 min. The person is asked to describe eight black and white drawings and to imagine what has preceded and what might follow in each. The sequence of images is given in a fixed order and is arranged to activate the attachment system gradually. The drawings depict situations that may recall attachment primal elements as loneliness, separation, death, illness, and abuse (i.e., attachment activators)^45^. In addition, the pictures also depict adult/adult, adult/child interactions inducing the subject to access the mental availability of attachment figures at different stages of life. Because the stimuli in the images are ambiguous, the subject must retrieve his or her own mental representations of attachment to create a narrative. The entire interview is recorded. Transcription and coding may follow if the goal is to identify attachment state of mind classifications groups. In this study, we used AAP exclusively to activate the neurobiological system of attachment before HBR stimulation.

#### HBR stimulation and recordings

HBR was elicited by transcutaneous electrical stimulation of the right median nerve at the wrist using a bipolar surface electrode (constant current square-wave pulses; DS7A, Digitimer). First, for each participant, we calibrated the stimulus intensity able to elicit a clear blink reflex by increasing the stimulus intensity until a clear and stable HBR was observed in at least five consecutive trials or the participant refused to increase the intensity further (mean stimulus intensities, 23.35 ± 17.48 mA; range, 0.7–75 mA). Stimulus duration was 200 μs and, to minimize habituation, the inter-stimulus interval was ~ 30 s, as described in the literature^6–9,16,51^. EMG activity was recorded from the right orbicularis oculi muscle (ipsilateral to the stimulated hand), using a pair of bipolar surface electrodes, with the active electrode placed over the mid lower eyelid and the reference electrode placed laterally to the outer canthus. Signals were amplified and digitized at 10 kHz (BIOPAC system, MP150), and stored for off-line analysis.

#### Experimental sessions

**Figure 1:**
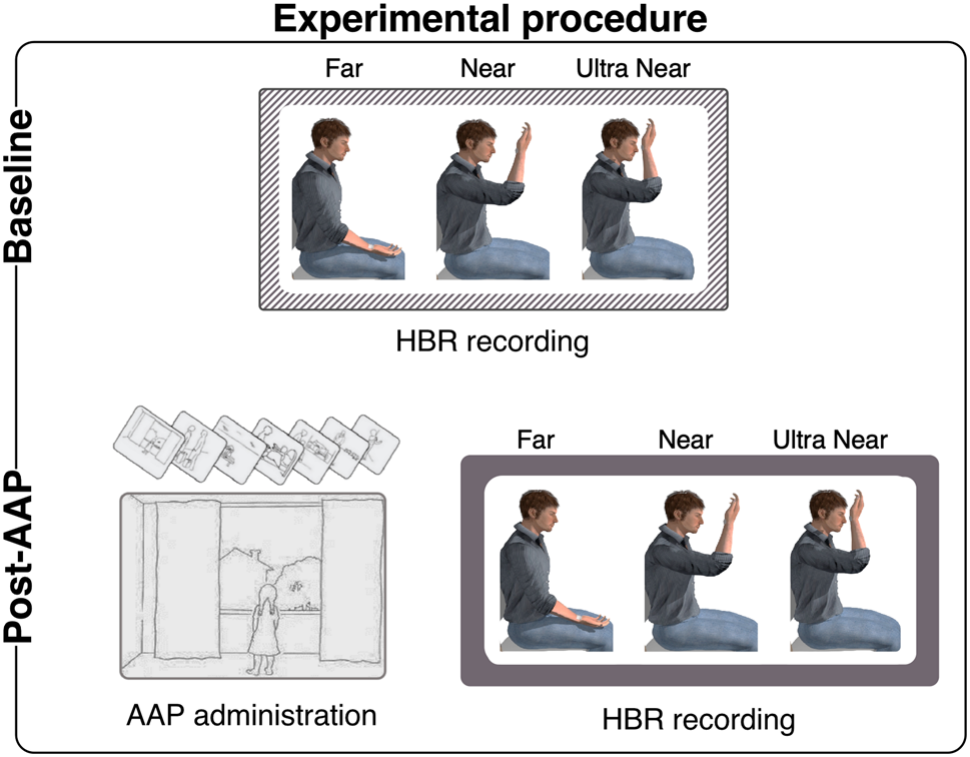
Experimental procedure. Participants underwent HBR recording in two experimental sessions that differed in whether or not they were immediately preceded by the administration of the AAP (Post-AAP session and Baseline, respectively). The images were created with the following software: Matlab R2020b (https://it.mathworks.com/products/matlab.html) and GraphPad Prism 8 (https://www.graphpad.com/scientific-software/prism/) were used to plot the data. Canvas Draw (https://www.canvasgfx.com/products/canvas-x-draw) was then used to combine all components of the figure into a single panel. The human model has been adapted from a previous study^14^.

On separate days from the AAI, participants underwent HBR recording in two experimental sessions that differed in whether or not AAP had been administered prior to HBR recording (Post-AAP session and Baseline, respectively). Note that the two sessions were acquired at 1 week of distance and the order was counterbalanced among participants. The Baseline session aimed to obtain a reference measure of HBR enhancement by the proximity of the hand to the face, which has been shown to be stronger the closer the hand is to the face^16^. The Post-AAP session aimed to test whether activation of the attachment system affects the magnitude of HBR enhancement. In both sessions, HBR was recorded while participants performed a postural manipulation task. They had to hold their stimulated hand in three different positions depending on how far away from their face they were: Far, Near, and Ultra-Near^8,16^. In the Far condition, participants sat with their forearm resting on their leg and their right hand close to the ipsilateral knee at a distance of ~ 60 cm from the ipsilateral side of their face. In the Near condition, participants sat holding their right arm with their wrist at a distance ~ 20 cm from the ipsilateral side of their face. In the Ultra-Near condition, participants sat and held their right arm with their wrist at a distance of ~ 4 cm from the ipsilateral side of their face (with fingers never touching the face), namely inside the PPS of the face (see Fig. 1). Prior to the start of the experiment, participants were instructed on where to place their hand in each position. On each trial, the experimenter called the hand position (i.e., Far, Near or Ultra-Near) and participants were required to place their right arm in the appropriate position. Each experimental session consisted of a total of twenty-four trials (eight in the Far position, eight in the Near position, and eight in the Ultra-Near position) performed in alternating trials. The interval between successive stimuli was about 30 s, thus each block lasted about 12 min.

#### State-trait anxiety inventory and beck depression inventory

To better explore the trend of HBR modulation and investigate whether individual differences in trait anxiety and depression predict HBR enhancement, we also administered two self-reports, namely the subscale for trait anxiety of the State-Trait Anxiety Inventory (STAI-T) and the Beck Depression Inventory (BDI-II). The STAI-T^57^ is a 20-item self-report measure commonly used to assess persistent anxiety traits. Respondents are asked to rate each item on a 4-point Likert scale. The total score ranges from 20 to 80, with higher scores indicating greater trait anxiety. The BDI-II^58^ consists of 21 items, each rated on a 4-point Likert scale, that assess the presence of depressive symptoms. The total score ranges from 0 (no depressive symptoms) to 63 (severe depression).

### Data preprocessing

EMG pre-processing and analysis were performed using Matlab (http://www.mathworks.com) and Letswave 6 (http://nocions.webnode.com) software^59^. EMG signals from each participant were high-pass filtered (55 Hz) and full-wave rectified. HBR responses were averaged separately by position, resulting in three average HBR waveforms for each session per participant (i.e., far, near, ultra-near).

### Data analysis

To analyse the EMG data, we extracted and measured the area under the curve (AUC) of each participant for each experimental session and hand position.

To examine the magnitude of HBR in each group of participants (i.e., organized and disorganized), we first used a Linear Mixed Model (LMM) approach as implemented in SPSS 28.0 (IBM, Chicago, IL, USA). We thus run LMM, three-way ANOVA with the HBR amplitude (i.e., AUC) as dependent variable. Fixed effect factors were ‘hand position’ (three levels: Far, Near, Ultra Near) and ‘session’ (two levels: Baseline, Post-AAP). Participants were considered as a random effect variable to properly use all observations for effect estimation. Post-hoc comparisons were performed using the Bonferroni test.

Based on the results of the within-group analysis showing a peculiar pattern of HBR modulation in the disorganized group, we then adopted two different analysis approaches to better investigate the differences between the groups.

In the first one, because the disorganized group showed a different pattern of HBR increase than the organized group, we calculated an index of position-dependent HBR increase expressed as a delta between HBR magnitude in the Near and Far positions (Near-Far) and between the Ultra Near and Near positions (Ultra Near-Near), regardless of session. The greater the position-dependent index the greater should be the difference between far and near and between near and ultra-near. Each index was compared between groups using an unpaired T-test (one-tailed).

The second approach aimed to better investigate the different pattern of HBR increase between the disorganized and organized groups in the two sessions. To this end, we calculated an index of AAP-dependent modulation of HBR magnitude expressed as a delta between the HBR magnitude in the Post-AAP session and that in the Baseline session (AAP-dependent index = Post-AAP session − Baseline session), regardless of hand position. The greater the AAP-dependent index the greater should be the difference between the two sessions. The values obtained were compared between groups using an unpaired T-test (one-tailed).

Pearson correlations were conducted between HBR enhancement indexes and individual scores on the STAI-T and the BDI-II to investigate the possible role of individual differences in trait anxiety and depression in HBR enhancement.

## Results

Sixteen (61.5%) and ten (38.5%) participants were classified as organized and disorganized, respectively, according to the AAI coding system^56^. The percentage of participants who were classified as disorganized is approximately double what would be expected in a non-clinical sample^53^. This is due to the fact that the sampling procedure was designed to increase the likelihood of using the AAI to interview individuals with a disorganized state of mind. This sampling procedure is described in detail elsewhere^60^.

**Figure 2:**
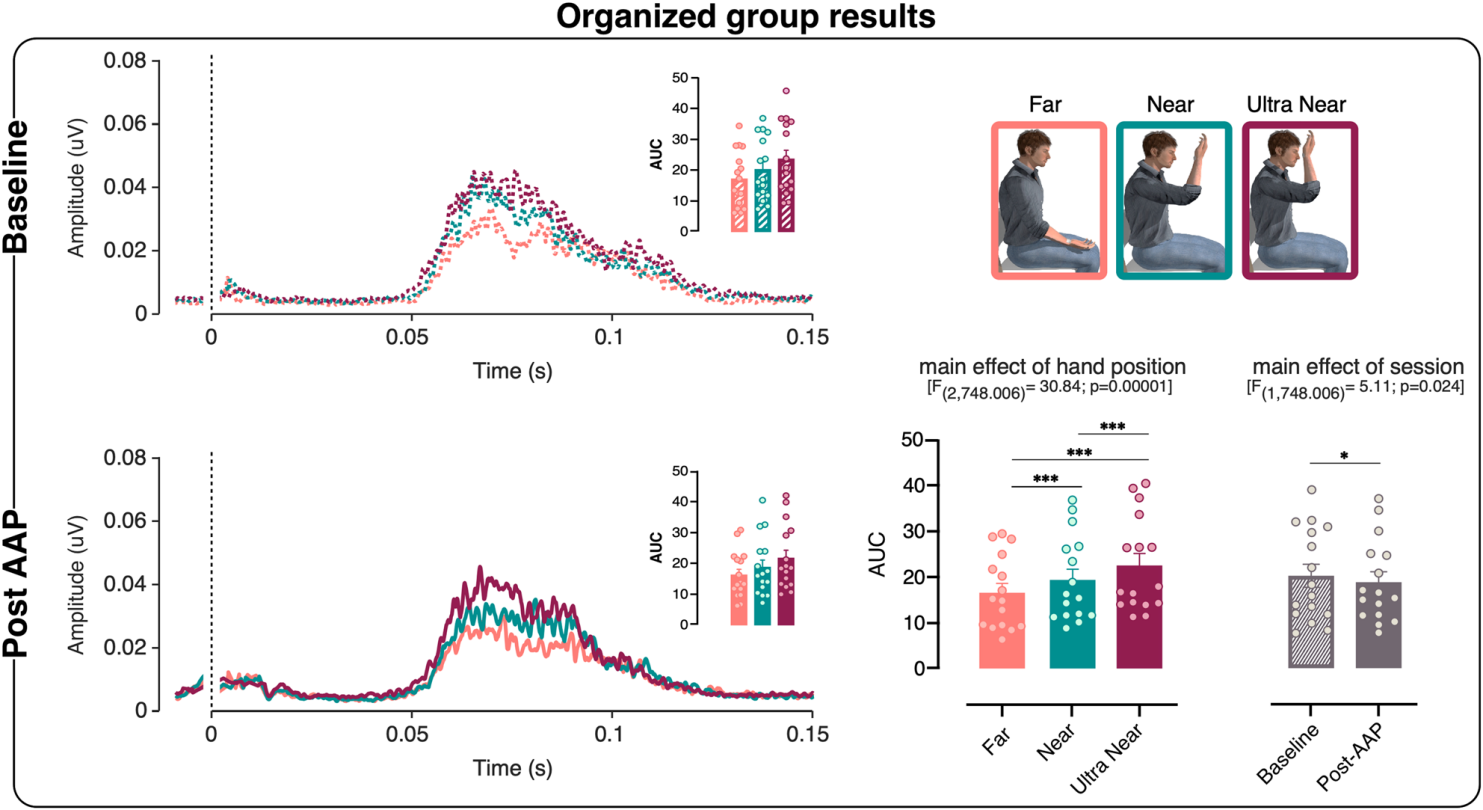
Organized group results. Left panels, rectified hand blink reflex (HBR) waveforms (group‐average) in the three different hand positions: Far (coral), Near (green), and Ultra Near (magenta). The dotted line at 0 s represents the stimulus delivery. The histograms represent the HBR magnitude (AUC) in the three hand positions. Bottom right panel, main effect of hand position. The error bars show the standard error of the mean (SEM) and the asterisk shows the significant difference (*p < 0.05). The dots represent individual participants. The images were created with the following software: Matlab R2020b (https://it.mathworks.com/products/matlab.html) and GraphPad Prism 8 (https://www.graphpad.com/scientific-software/prism/) were used to plot the data. Canvas Draw (https://www.canvasgfx.com/products/canvas-x-draw) was then used to combine all components of the figure into a single panel.

The LMM performed on the HBR magnitude of the organized group (N = 16) shows a significant main effect of hand position [F_(2,748.006)_ = 30.839; p = 0.00001], indicating a linear trend in HBR increase regardless of the experimental session, with a significantly greater HBR magnitude in both Near (p = 0.00001) and Ultra Near (p = 0.000001) positions as compared to the Far position, and a significantly greater magnitude in the Ultra Near than in the Near position (p = 0.00001). Furthermore, we observe a significant main effect of session [F_(1,748.006)_ = 5.108; p = 0.024], suggesting that the overall magnitude of HBR was greater in the Baseline session than in the post-AAP session, regardless of the position of the hand in space (see Fig. 2).

**Figure 3:**
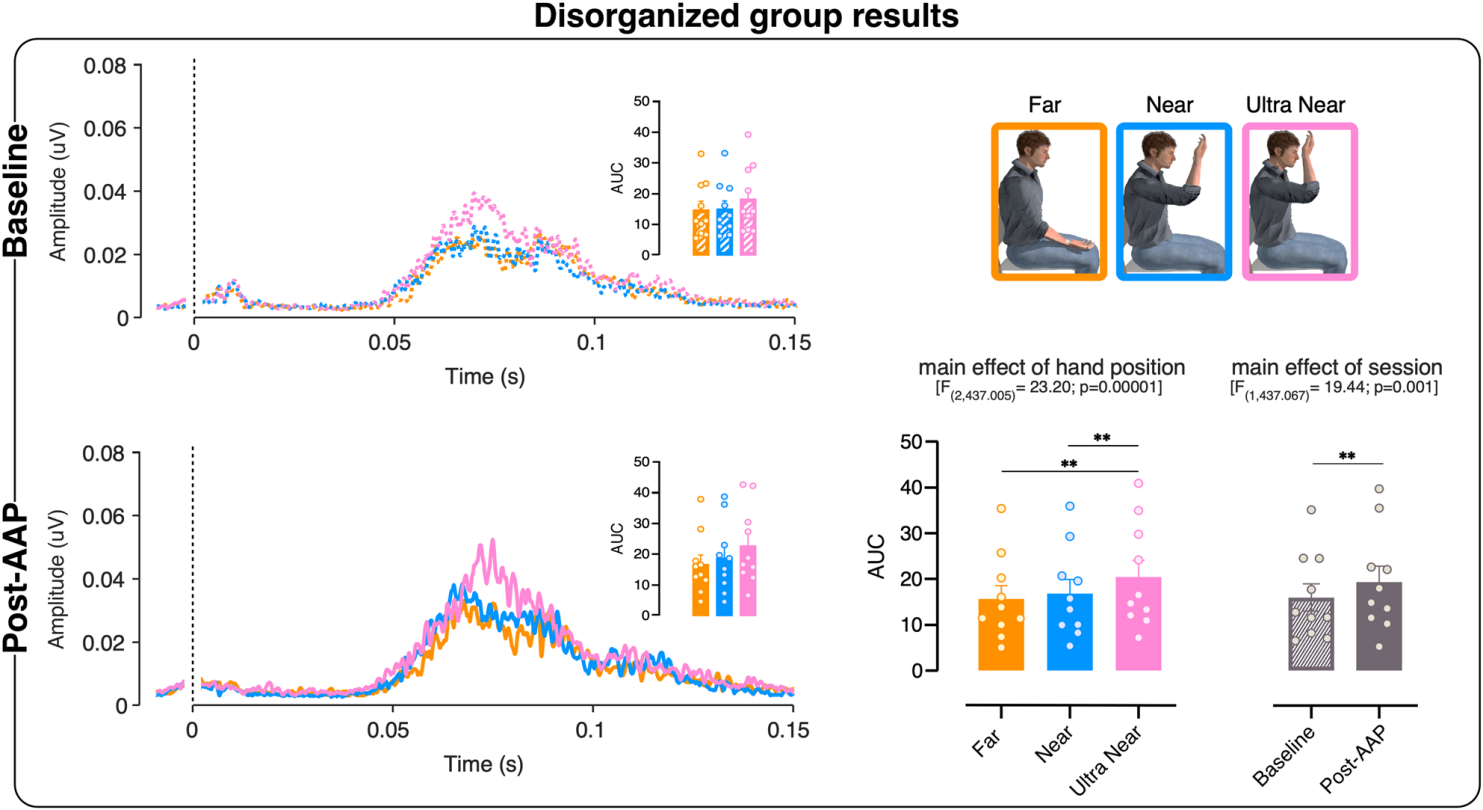
Disorganized group results. Left panels, rectified hand blink reflex (HBR) waveforms (group‐average) in the three different hand positions: Far (orange), Near (light blue), and Ultra Near (pink). The dotted line at 0 s represents the stimulus delivery. The histograms represent the HBR magnitude (AUC) in the three hand positions. Bottom right panel, main effect of hand position and main effect of session. The error bars show the standard error of the mean (SEM) and the asterisk shows the significant difference (*p < 0.05). The dots represent individual participants. The images were created with the following software: Matlab R2020b (https://it.mathworks.com/products/matlab.html) and GraphPad Prism 8 (https://www.graphpad.com/scientific-software/prism/) were used to plot the data. Canvas Draw (https://www.canvasgfx.com/products/canvas-x-draw) was then used to combine all components of the figure into a single panel.

In the disorganized group (N = 10), the LMM also shows a significant main effect of hand position [F_(2,437.005)_ = 23.202; p = 0.00001]. Post-hoc comparisons show that HBR increase is significantly greater in the Ultra Near position than in the Far (p = 0.001) and the Near (p = 0.001) positions, while no difference emerged between Far and Near positions (p = 0.33). Furthermore, we observe a significant main effect of session [F_(1,437.067)_ = 29.885; p = 0.001], suggesting that the overall magnitude of HBR is greater in the Post-AAP session compared to the Baseline session, regardless of the position of the hand in space (see Fig. 3).

**Figure 4:**
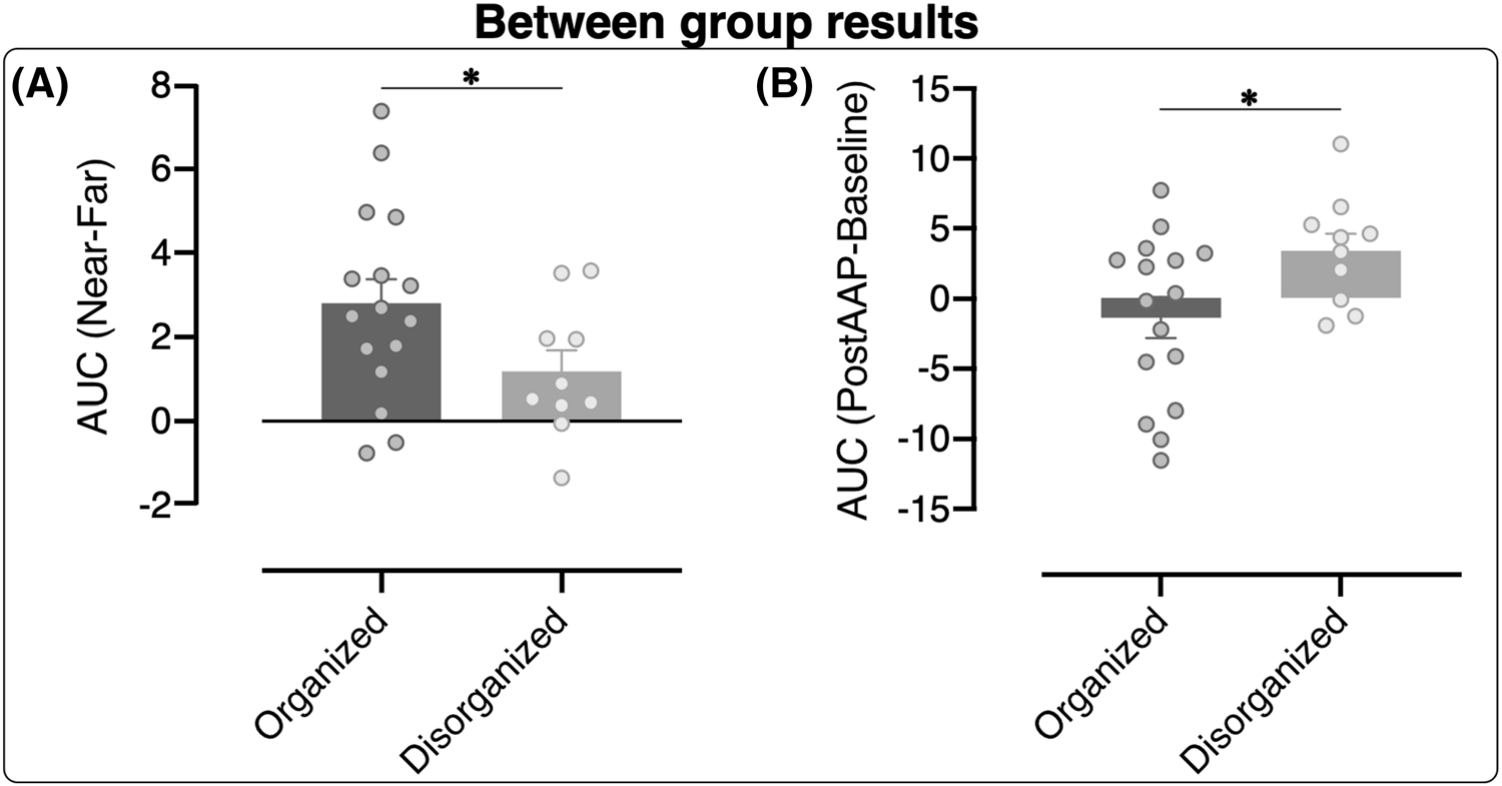
Between group results. (**A**) HBR position-dependent index expressed as Near-Far in the organized (dark grey) and disorganized (light grey) groups. (**B**) HBR AAP-dependent index expressed as AAP-Baseline in the organized (dark grey) and disorganized (light grey) groups. The error bars indicate standard error of the mean (SEM) and asterisk indicates the significant difference (*p < 0.05). The dots represent individual participants. The images were created with the following software: Matlab R2020b (https://it.mathworks.com/products/matlab.html) and GraphPad Prism 8 (https://www.graphpad.com/scientific-software/prism/) were used to plot the data. Canvas Draw (https://www.canvasgfx.com/products/canvas-x-draw) was then used to combine all components of the figure into a single panel.

The between-group analysis performed on the position-dependent HBR indexes shows that the two groups differ only in the Near-Far index (t_24_ = 1.954; p = 0.0312; dz = 0.82) and not in the Ultra Near-Near index (p = 0.323). This suggests that, regardless of the experimental session, only organized participants show a significant HBR increase in the Near position compared to the Far position, while disorganized participants do not (Fig. 4A).

The between-group analysis performed on the AAP-dependent HBR index shows a significant difference between groups (t_24_ = 2.256; p = 0.0167; dz = 0.95), indicating that HBR increase between Baseline and Post-AAP session was significantly greater in the disorganized group than in the organized group, regardless of hand position (see Fig. 4B).

As for the correlation analyses, no significant results were obtained (always p > 0.05).

## Discussion

In recent years, there has been a growing body of literature demonstrating the medium- and long-term effects of disorganizing attachment experiences in childhood on various aspects of mental functioning. For example, a disorganized state of mind has been associated with high-level alterations in integrative mental functioning, leading to impairments in cognitive, metacognitive and emotional regulation^40,41,61,62^. It has been suggested that activation of attachment memories in individuals with disorganized state of mind may act as a trigger for disintegrative processes that impair optimal functioning of higher-level mental functions in similar ways to other trauma-related pathogenic processes involving executive and regulatory functions, self-related processes, social cognition and mentalization^40,61–64^. Given that the neurobiological system of attachment has been described as part of a hierarchically organized control system in which certain neural structures such as the orbitofrontal cortex, anterior cingulate and amygdala can exert top-down regulation on brainstem activity that mediates defensive PPS, one of the main aims of our work was to investigate whether the effects of disorganized attachment on higher-level mental functions already described in the literature also extend to a more automated and sub-cortical driven aspect such as defensive PPS. To do this, we used the opportunity provided by the AAP to activate the attachment system and measured the patterns of HBR responses in individuals with disorganized IWM.

Our findings replicate the HBR enhancement induced by the proximity of the hand to the face, as widely described in previous studies^6–9,11,14,16,51^, nicely confirming that the closer the threatening hand is to the face, the stronger the participants’ defensive responses are. In line with our hypothesis, our new finding is that the participants’ mental state of attachment can have a great influence on the modulation of such defensive response. Indeed, a peculiar pattern of HBR enhancement in the disorganized group emerged from both the within analysis and the between group analysis.

In particular, the group of organized participants showed a linear trend in HBR enhancement, with HBR increasing the closer the hand to the face, regardless of the experimental session. In contrast, the group of disorganized participants shows a peculiar pattern of modulation of HBR enhancement. Specifically, participants with disorganized IWM do not show a linear trend in HBR magnitude as a function of hand proximity. In fact, they show an abrupt enhancement in the Ultra-Near position compared to the Far and Near position. This suggests that the extent of their defensive PPS boundaries is limited to the space immediately around the face and is not gradually shaped by the proximity of the hand to the face.

As for the comparison between the Baseline and the Post-AAP sessions, the two groups show an inverse pattern. The organized group is characterised by an overall greater HBR magnitude in the Baseline session compared to the post-AAP session, while the disorganized group has a significantly overall greater HBR magnitude in the Post-AAP session compared to the Baseline session. This suggests that the triggering of emotional and relational memories of early attachment experiences by the AAP leads organized participants to experience the experimental session as less threatening than the Baseline session, while in the disorganized group the triggering of these memories leads participants to experience the Post-AAP session as more threatening than the Baseline one.

We can consider the defensive pattern of the organized group as the most adaptive response to threats. Indeed, their defensive response is gradually enhanced the closer the threats are (i.e., linear trend in HBR enhancement), suggesting that their defensive system defines the boundaries of the potential high-risk area of their surrounding space as more extended, leading them to respond to threats at a greater distance. In contrast, our data show an alteration of the defensive pattern in the disorganized group. Indeed, they show an abrupt HBR enhancement only at a closer distance, suggesting that the boundaries of their defensive cloud are smaller. Their defensive pattern thus seems to be less adaptive. Given the evolutionary importance of responding to potential threats in space, having a mechanism for encoding space very early in life seems to be beneficial^65^. However, the ability to gradually shape the defensive response to threats as a function of distance is likely to be built up over time and through experience. Therefore, we can speculate that early attachment experiences have an impact on how the boundaries of the representation of the defensive PPS are encoded. Thus, the frightening and threatening parental behaviour associated with disorganized IWM, leading to impairments in cognitive, metacognitive and emotional regulation, could also prevent the boundaries of the defensive PPS from being properly set in an adaptive manner. The reverse pattern observed in the overall magnitude of the HBR also seems to confirm the regulatory role of early attachment experiences in the development and functioning of the defensive PPS. In fact, when the neurobiological attachment system is activated, the presence of organized IWM seems to be a resource in situations where it is necessary to cope with a threat, while, on the contrary, disorganized IWM lead to an increase in threat perception, precisely because it was probably the very frightening and threatening parental behaviour that disorganized the IWM and consequently the defensive PPS.

The present study has some limitations that should be considered. Although we enrolled a number of participants consistent with previous works, further studies with a larger number of participants are needed to confirm the current results. In addition, although there is ample evidence of which brain areas are involved in the phenomena we investigated, we have no direct evidence of the effects of our experimental task on the functioning of these brain areas. Future works should replicate our study by using the same experimental procedure and employing appropriate methods to obtain direct data on the areas that are actually activated during performance of our task.

Despite the limitations described above, our results support the hypothesis that a defensive PPS exists and show for the first time that this space and associated defensive responses are significantly modulated by individual mental representations related to early attachment experiences. Interestingly, a relationship between disorganized attachment-related state of mind and psychopathology^66^ and between psychopathology and PPS has been suggested^67^. The fact that in the present work we found a modulation of defensive PPS in a nonclinical sample makes this finding particularly relevant because it underscores the crucial role that high-level attachment-related mental representations play in organising PPS and associated subcortical responses, whether or not these responses are manifested by an individual with a clinical profile. This may imply that it is not psychopathology per se that alters the defensive PPS but the fact of having developed disorganized IWM. Future work should take this into account and further investigate the causal role of disorganized attachment-related state of mind in shaping the defensive PPS and the conditions under which such disorganization can sometimes determine the development of psychopathological profiles characterized by bodily self-consciousness alterations.

## Data Availability

Data are available from the first author (C.F.) upon request.
